# Addiction, attachment, and the brain: a focused review of empirical findings and future directions

**DOI:** 10.3389/fnhum.2025.1625880

**Published:** 2025-06-19

**Authors:** Human-Friedrich Unterrainer

**Affiliations:** ^1^Faculty of Psychotherapy Science, Sigmund Freud Private University, Vienna, Austria; ^2^Department of Religious Studies, University of Vienna, Vienna, Austria; ^3^Center for Integrative Addiction Research (CIAR), Grüner Kreis Association, Vienna, Austria; ^4^Department of Psychiatry and Psychotherapeutic Medicine, Medical University of Graz, Graz, Austria

**Keywords:** addiction, attachment, developmental neuroscience, interoception, insular cortex, EEG neurofeedback, psychodynamic therapy, early trauma

## Abstract

This focused review integrates theoretical and empirical work from developmental neuroscience, attachment theory, and psychodynamic psychotherapy to reconceptualize addiction as a disorder rooted in disrupted attachment and altered brain function. Drawing on both clinical and research findings, it explores how early relational trauma contributes to dysregulation of stress-response systems and functional changes in brain regions involved in self-awareness, emotion regulation, and reward processing. Particular attention is given to the insular cortex and its role in interoception as it relates to addictive behavior. EEG neurofeedback is introduced as an emerging therapeutic tool, illustrated through a clinical case study that demonstrates how its combination with psychodynamic therapy can foster both neurophysiological regulation and emotional insight. This work supports a view of addiction as a disconnection from bodily and relational signals, rooted in early attachment experiences, and contributes to a more integrative, developmentally informed treatment model.

## 1 Introduction

Addiction is now widely recognized as a condition emerging from a complex interplay of biological, psychological, and social factors. The World Health Organization’s early definition (1964) remains influential, describing addiction as a state of periodic or chronic intoxication caused by repeated use of natural or synthetic substances, which is harmful to both the individual and society (as cited in Möller et al., 2015). Contemporary perspectives have expanded this definition to encompass behavioral addictions as well. Factors such as substance availability, individual vulnerabilities, and environmental influences-including family dynamics, peer relationships, and life stressors-interact to shape an individual’s trajectory toward substance use and addiction (Volkow et al., 2016).

The idea that addiction may stem from disrupted attachment patterns dates back to Sigmund Freud. Although Freud did not formulate a comprehensive theory of addiction, he observed that removing the substance alone was insufficient for successful treatment (Freud, 1898). His broader conceptualization of sexuality and relational drives aligns with modern psychodynamic and attachment-oriented models, which emphasize the importance of personality structure and developmental history in understanding addiction (Zellner et al., 2011). In line with this, Orford (2001) conceptualized addiction as a form of excessive appetite and behavioral fixation—an intense attachment to an activity that becomes uncontrollable despite adverse consequences. Similarly, Khantzian (2013) described addiction as a maladaptive strategy for emotional self-regulation, reflecting deeper disturbances in personality organization.

Attachment theory, originally developed by Bowlby and Ainsworth (Bretherton, 2013), posits that early relational experiences shape enduring internal working models of self and others. These models influence affect regulation and interpersonal functioning throughout life. Ainsworth’s Strange Situation Test (Ainsworth et al., 2015) identified four attachment patterns: secure, insecure-avoidant, insecure-ambivalent, and disorganized. Disorganized attachment—often linked to early trauma and inconsistent caregiving—is particularly relevant to later psychopathology, including substance use disorders (see Mikulincer and Shaver, 2012 for an in-depth discussion).

## 2 Addiction as an attachment disorder

In the postscript to Attachment Across the Life Cycle, John Bowlby observed, “Once we postulate the presence within the organism of an attachment behavioral system regarded as the product of evolution and as having protection as its biological function, many of the puzzles that have perplexed students of human relationships are found to be soluble” (Parkes et al., 2006, p. 293). This evolutionary framework supports the proposition that addiction can be conceptualized as an attachment disorder—particularly when viewed through the lens of relational trauma and disrupted affect regulation.

Our empirical findings support this interpretation. In a study conducted within a long-term therapeutic community setting, individuals with substance use disorders (SUD) exhibited elevated levels of insecure attachment and traits associated with borderline personality organization (Hiebler-Ragger et al., 2016). A follow-up study found that while patients with insecure attachment were initially less likely to drop out of therapy, their trust in self and others—an indicator of secure attachment—tended to decline during early treatment (Fuchshuber et al., 2018). This may reflect a transition from initial idealization of the therapeutic environment to a more realistic appraisal, which could facilitate more adaptive therapeutic engagement.

Conversely, other findings revealed a negative association between insecure attachment and both therapy motivation and therapeutic alliance in similar populations (Rübig et al., 2021). Additionally, data from a non-clinical sample showed a negative correlation between disorganized attachment and the use of sedatives and opioids (Fuchshuber et al., 2025b). These mixed results underscore the complex and context-dependent role of attachment in the etiology of addiction and in shaping treatment responsiveness (Fuchshuber and Unterrainer, 2020).

## 3 A neuro-evolutionary view of attachment and addiction

From a neuro-evolutionary perspective, attachment is understood as a biologically rooted motivational and behavioral system shaped by early interactions with caregivers. This system is closely linked to affect regulation, social bonding, and neurobiological development (Schindler, 2019). Feldman (2017) identified distinct neural circuits that underlie familial, romantic, and platonic attachments, each engaging specific affective and cognitive processes. Our work has been strongly influenced by Jaak Panksepp’s theory of primary emotional systems (Panksepp and Biven, 2012), which include SEEKING, LUST, CARE, ANGER, FEAR, PANIC, and PLAY. These systems originate in subcortical brain structures such as the brainstem and periaqueductal gray (PAG), and they shape more complex emotional experiences—such as empathy, guilt, or shame—that are processed in neocortical regions associated with mentalization and mindfulness.

**Figure 1:**
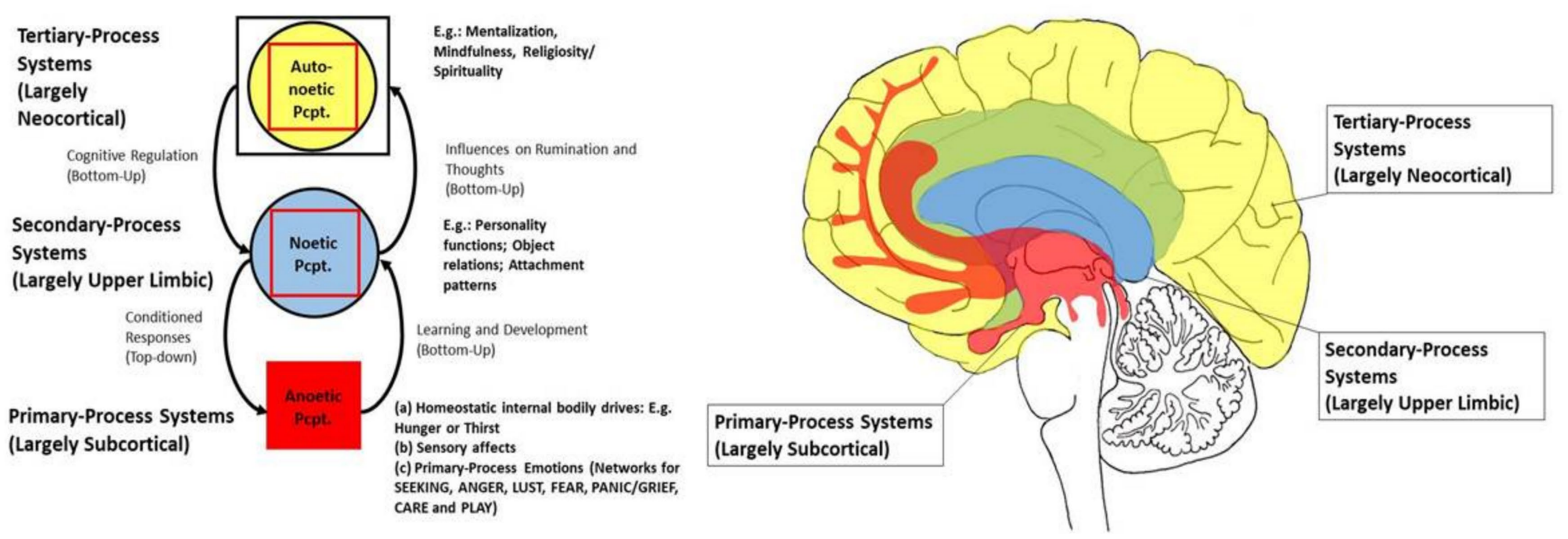
The triune brain model [based on Panksepp and Biven, 2012, Solms and Panksepp, 2012, and Kernberg, 2015]; Original sketch drawn by Jürgen Fuchshuber (slightly adapted; used with kind permission); Pcpt., perception.

Although Mac Lean (1990) triune brain model has been criticized for oversimplification (Barratt, 2015), it remains a useful heuristic for conceptualizing the relationships between emotion, personality, and cognition (see Figure 1 for further illustration). In our own studies, we used behavioral data to explore how Big Five personality traits mediate the relationship between primary emotional systems and religious-spiritual well-being. We found that CARE (positively) and ANGER (negatively) predicted spiritual well-being. When personality traits were included as mediators, these effects diminished, while extraversion and agreeableness emerged as significant predictors (Hiebler-Ragger et al., 2018). To address a conceptual gap, we developed a German-language LUST scale and integrated it into our version of the Brief Affective Neuroscience Personality Scales (BANPS–GL; Fuchshuber et al., 2022, 2023).

**Figure 2:**
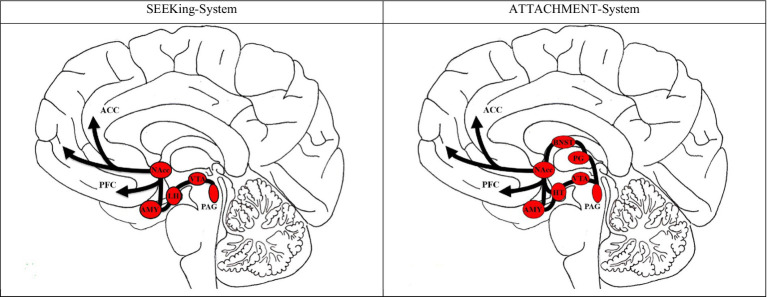
Neural correlates of SEEKing versus Attachment (social bonding) in the brain. ACC, anterior cingulate cortex; PFC, prefrontal cortex; NAcc, nucleus accumbens; AMY, amygydala; LH, left hypothalamus; VTA, ventral tegmental area; PAG, periaqueductal gray; BNST, bed nucleus of the stria terminalis; PG, pineal gland. Original Sketch drawn by Jürgen Fuchshuber; slightly modified; used with kind permission; based on Coan (2010), Panksepp (2008), and Panksepp and Biven (2012).

Animal and human studies alike show that similar neural circuits are activated during SEEKING and social bonding, particularly involving dopaminergic pathways connecting the amygdala and nucleus accumbens to the prefrontal cortex (Barrett et al., 2013; Coan, 2010; see Figure 2 for further illustration).

In our neuroimaging research, patients with poly-drug use disorder (PUD) showed structural brain abnormalities and impairments in attachment and personality organization compared to healthy controls (Unterrainer et al., 2016, 2019). Behavioral difficulties in emotion regulation were consistent across studies, though expected neural correlates were less pronounced. For instance, during an emotion reappraisal task in an fMRI scanner, PUD patients displayed emotional dysregulation, yet anticipated neural activation patterns were attenuated (Hiebler-Ragger et al., 2021). Resting-state fMRI analyses revealed altered connectivity between the default mode network (DMN) and salience network in PUD patients—particularly among those with insecure attachment patterns (Fuchshuber et al., 2025a). These findings suggest a neurobiological basis for impaired affective processing and social cognition in individuals with addiction and attachment disturbances.

We also investigated the role of oxytocin, a neuropeptide implicated in bonding. Using attachment-related stimuli from the Adult Attachment Projective (AAP) system (George and West, 2012), we measured oxytocin responses in PUD patients versus controls (Fuchshuber et al., 2020). While behavioral responses did not differ significantly between groups, oxytocin reactivity was significantly blunted in the addiction group. This suggests reduced physiological sensitivity to attachment cues and highlights a possible neurobiological mechanism underlying impaired relational functioning in addiction. It also raises the question of whether pharmacological substitution therapies might affect the attachment system in ways that hinder interpersonal engagement in treatment.

## 4 Clinical implications and future perspectives

The conceptualization of addiction as an attachment disorder is exemplified in a case study involving a 19-year-old college student treated with a combined approach of EEG neurofeedback and psychodynamically oriented psychotherapy (Unterrainer et al., 2014). The patient presented with depressive symptoms following a period of polysubstance use. Across 11 treatment sessions, including a follow-up, clinical progress was assessed using the Montgomery-Åsberg Depression Rating Scale (MADRS; Montgomery and Åsberg, 1979), the Brief Psychiatric Rating Scale (BPRS; Overall and Gorham, 1962), and the Beck Depression Inventory (BDI; Beck et al., 1996). These measures showed a clear shift from clinically significant depression to a stable, non-pathological state—confirmed at follow-up.

Neurophysiologically, improvements were mirrored by increased activity in the sensorimotor rhythm (SMR)/theta ratio, highlighting the potential utility of EEG neurofeedback. Equally significant was the strong therapeutic bond formed between the patient and the multidisciplinary treatment team. The team’s diversity—comprising an Austrian addiction therapist, a Taiwanese neurofeedback trainer, and an English neuropsychologist—may have contributed to the development of a meaningful male attachment figure for the patient (see Unterrainer et al., 2013 for further discussion). These results align with other studies from our group demonstrating the effectiveness of EEG neurofeedback in treating addiction (Lackner et al., 2016a; Unterrainer et al., 2013), as well as in the treatment of anorexia nervosa (Lackner et al., 2016b). More broadly, our findings reinforce a growing consensus that addiction is deeply interwoven with disruptions in attachment, emotion regulation, and personality structure (Unterrainer et al., 2018).

Future research should continue to build on this integrative framework by examining neuroplastic changes associated with long-term addiction therapy—particularly within attachment-based psychotherapeutic approaches. Given the established links between insecure attachment and vulnerability to substance use (Fairbairn et al., 2018; Schindler, 2019), and the potential of such therapies to modulate neural circuits involved in reward, stress, and social bonding (Lewis et al., 2020), longitudinal neuroimaging studies are needed. These investigations could illuminate how recovery processes manifest at the neurobiological level, informing the development of more targeted, personalized interventions that address both relational and neurophysiological dimensions of addiction recovery.
